# Supplementary material for: Development and Experimental Validation of a Novel Prognostic Signature for Gastric Cancer
Source: Cancers (Basel). 2023 Mar 5;15(5):1610. doi: 10.3390/cancers15051610 (PMC10000504; doi:10.3390/cancers15051610)
Supplement: Supplementary file 1 [file cancers-15-01610-s001.zip › Figure legends_right version.pdf]

Supplementary Figure legends

Figure S1. (A) Bubble plot of GO enrichment for GC in the cardiac zone, the fundus/corpus zone, and the pyloric zone. Green represents biological processes; red represents cellular components; blue represents molecular functions. (B) Identify differentially expressed genes (DEGs) and KEGG enrichment of DEGs for GC in the cardiac zone, the fundus/corpus zone, and the pyloric zone. The color of the bubble represents the significance of p.adjust. (C) Analysis of network topology for different soft-threshold power. The left panel shows the impact of soft threshold power on the scale-free topology fit index; the right panel displays the impact of soft-threshold power on the mean connectivity.

Figure S2. GO and KEGG enrichment of genes in the purple, brown and salmon modules. (A)-(C) GO enrichment of the purple module (correlated with the cardiac zone) (A), salmon module (correlated with fundus/corpus zone) (B) and brown module (correlated with the pyloric zone) (C). (D)-(F) KEGG enrichment of the purple module (correlated with the cardiac zone) (D), salmon module (correlated with the cardiac zone) (E) and brown module (correlated with the pyloric zone) (F).

Figure S3. Survival analysis and binary classification of the genes from the co-expression networks (A) Kaplan-Meier curves of OS according to the top5 genes (APOA4, MS4A10, SLC28A1, AQP10, APOB) from the cardiac zone co-expression network separate of GC in the cardiac zone (left, p=0.074), the fundus/corpus zone (middle, p=0.22), and the pyloric zone(right, p=0.73) in TCGA target GTEx. (B) Kaplan-Meier curves of OS according to the top5 genes (APOA4, MS4A10, SLC28A1, AQP10, APOB) from the cardiac zone co-expression network separate of GC in the cardiac zone (left, p=0.059), the fundus/corpus zone (middle, p=0.15), and the pyloric zone(right, p=0.33) in GSE66229. (C) Kaplan-Meier curves of OS according to the top5 genes (VCAN, COL1A2, FAP, PODNL1, SULF1) from the fundus/corpus co-expression network separate of GC in the cardiac zone (left, p=0.16), the fundus/corpus zone (middle, p=5.32e-6), and the pyloric zone(right, p=0.73) in TCGA target GTEx. (D) Kaplan-Meier curves of OS according to the top5 genes (VCAN, COL1A2, FAP, PODNL1, SULF1) from the fundus/corpus co-expression network separate of GC in the cardiac zone (left, p=0.83), the fundus/corpus zone (middle, p=0.0047), and the pyloric zone(right, p=0.045) in GSE66229. (E)-(F) BoxPlot of AUC of two classifiers (LR, Logistic Regression; RF, Random Forest) with the top5 genes (APOA4, MS4A10, SLC28A1,

AQP10, APOB) and the top5 genes (VCAN, COL1A2, FAP, PODNL1, SULF1) from the cardiac zone co-expression network(E) and the fundus/corpus co-expression network(F) separately as features in TCGA target GTEx and GSE66229. The cardiac zone means GC in the cardiac zone; the Fundus/Corpus zone means GC in the fundus/corpus zone; the Pyloric zone means GC in the pyloric zone.

Figure S4. (A) In the TCGA target GTEx cohort (383 GC samples with clinical information), the optimal λ was determined when the partial likelihood deviance reached the minimum value and further generated Lasso coefficients of the most useful prognostic genes. Data are presented as mean ± 95% confidence interval [CI]. (B) Cell-type markers. The expression levels of cell-type markers across cell types are shown. Cell-type marker genes were identified in Wilcoxon rank-sum test (FDR <0.01, and fold change >1.5) and only the top genes are shown in the figure. (C) The UMAP plot of 200,954 cells to visualize cell-type clusters. (D)The UMAP plot of both samples. (E)The UMAP plot of both cells from normal and tumor tissues(blue means tumor tissues; red means normal tissues).

Figure S5. (A) The UMAP plot of cell types marked by specific marker genes. The epithelial cell is marked by CDH1, and including pit mucous cell(Pit, MUC5AC and TFF1 positive), chief cell(Chief, LIPF and PGA3 positive), and intestinal cell(Intestinal, REG4 and TFF3 positive). Fibroblast is FN1, LUM, DCN positive; Pericytes is RGS5 and NOTCH3 positive; Endothelial cell is PLVAP and ACKR positive; T cell is CD8A positive; T regulatory cell(Treg) is IL2RA positive; NK cell is KLRD1 positive; B cell is MS4A1 positive; Plasma is TNFRSF 17 positive; mast cell is KIT positive; Macrophage is CD163 positive; Dendritic cell is PLD4 positive. (B) The UMAP plot of PRGS expression level.

Figure S6. (A)-(C) The UMAP plot of all samples(A), normal samples(B), tumor samples(C) according to PRGS score. (D) BoxPlot of PRGS in different cell types. Normal, normal samples; tumor, tumor samples; Diffuse, diffuse-type gastric cancer; Intestinal, intestinal-type gastric cancer; Mixed, mixed-type gastric cancer.

Figure S7. (A) Genome characterization and enrichment analysis. (A) Distribution map of transcription factors and TSS. (B) location distribution of Peaks on the genome. (C) relative proportions of gene coding regions, intergenic regions, introns, exons, and upstream and downstream regions. (D) Browser showing chromatin accessibility status of PRGS signature

(APOB, VCAN, ABCA6, CTSF). The grey bars highlighted peaks of PRGS signature in promoter regions. All of the genes showed duplication of two samples.

Figure S8. (A) Motif enrichment analysis of common genes around open chromatin regions. Fra1 and Foxa2 were found to be significantly enriched with APOB; KLF6 and Erra were found to be significantly enriched with VCAN; Fos and GABPA were found to be significantly enriched with ABCA6; BATF and Sp5 were found to be significantly enriched with CTSF (B)-(D) GO enrichment of the GC with ATAC-seq data. (E)KEGG enrichment of the GC with ATAC-seq data.

Figure S9. (A) Establishment of PRGS model dividing patients into high- and low- groups in the TCGA target GTEx(left), GSE66229(middle) and GSE15459(right). The cutoff value on the top (red: high expression; blue: low expression); survival statues on the bottom. (B)-(D) Kaplan–Meier curves of overall survival (OS) according to the PRGS of the different Lauren/WHO histotypes patients in TCGA target GTEx(B), GSE15459(C), and GSE66229(D). Intestinal means Intestinal-type gastric cancer patients; Diffuse means diffuse-type gastric cancer patients; Adenocarcinoma means gastric adenocarcinoma patients, and accurate histotypes is unknown; Tubular adenocarcinoma means tubular adenocarcinoma patients.

Figure S10. (A) Survival analysis of the different TNM stages patients. (A)-(F) Kaplan–Meier curves of overall survival (OS) according to the PRGS of the different TNM stages patients in TCGA target GTEx (A)-(C), and GSE66229 (D)-(F).

Figure S11. The integrated sankey diagram portrays the underlying correlations across the PRGS group, stage and OS status in the TCGA target GTEx cohort (345 samples with information on the clinical stage).

Figure S12. Multivariate Cox analysis evaluating independently predictive ability of PRGS model and other clinical characteristics for OS. (A) Multivariate Cox regression of PRGS regarding OS in TCGA target GTEx (345 samples with information on the clinical stage). (B) Multivariate Cox regression of PRGS regarding OS in GSE66229 (n=298). (C) Multivariate Cox regression of PRGS regarding OS in GSE15459 (n=191). Statistic tests: two-sided Wald test. Data are presented as hazard ratio (HR) ± 95% confidence interval [CI]. * $p < 0.05$. ** $p < 0.01$. *** $p < 0.001$.

Figure S13. The performance of PRGS was compared with other clinical and molecular variables in predicting prognosis. Statistic tests: two-sided z-score test.

Figure S14. (A) Heatmap showed that the expression level of cluster1 is higher than cluster2 in

GSE66229. (B) The consensus score matrix of all samples in GSE66229 when k = 3 or 4. (C) Kaplan–Meier analysis showed that patients in Cluster 1 exhibited better OS in the GSE66229 cohort when k=3(left, p=0.048) or k=4(right, p=0.0071). (D) Heatmap showed that the expression level of cluster1 is lower than others in GSE66229 when k=3(left) or k=4(right). (E) Heatmap showed that the expression level of cluster1 is higher than cluster2 in GSE15459. (F) The consensus score matrix of all samples in GSE15459 when k = 3 or 4. (G) Kaplan–Meier analysis showed that patients in Cluster 1 exhibited better OS in the GSE15459 cohort when k=3(left, p=0.00064) or k=4(right, p=0.00064). (H) Heatmap showed that the expression level of cluster1 is lower than others in GSE15459 when k=3(left) or k=4(right). C1 means cluster1, C2 means cluster2, C3 means cluster3, C4 means cluster4.

Figure S15. Evaluation of the PRGS signature with machine learning classifiers in GSE66229. (A)-(B) ROC curve of binary classification with logistic regression classifier(A) and random forest classifier(B) for all and different stage(stageI-IV) GC patients with PRGS signature as features. “Tumor” means GC patients; “Normal” means gastric tissues with a non-tumor state.

Figure S16. (A) BoxPlot of AUC in two classifiers (LR, Logistic Regression; RF, Random Forest), respectively. (B)-(D) HE staining of advanced gastric cancer (AGC) (B), normal tissue (C) and early gastric cancer (D).

Figure S17. Tumor mutation status of GCscore (FANCA, DUSP3, HIST1H3B, CLNS1A, FANCC) [6] and Kaplan–Meier curves of high frequency mutant genes. (A) Kaplan-Meier curves of OS according to the GCscore[6] in TCGA. (B) OncoPlot of significantly mutated genes in high- (left) and low- (right) GCscore groups. The mutation types with their frequencies were presented. (C)-(G) Kaplan–Meier curves of OS between the oncogenic mutation and wild type (WT) of MUC16(C), CSMD1(D), FAT4(E), TP53(F) and TTN(G).

Figure S18. Tumor mutation status of high- and low- PRGS groups. (A)The mutation percentages of PRGS signature (APOB, VCAN, ABCA6, CTSF) in high-(left) and low-(right) PRGS groups. (B) Interaction effect of genes mutating differentially in patients in the high-(left) and the low-(right) PRGS groups. (C) The mutation percentages of nine common oncogenes signaling pathways in high-(left) and low-(right) PRGS groups. (D) BoxPlot of nine common oncogenes signaling pathways expression profile in the high-(red) and low-(blue) PRGS groups.
