# Supplementary material for: Development and Experimental Validation of a Novel Prognostic Signature for Gastric Cancer
Source: Cancers (Basel). 2023 Mar 5;15(5):1610. doi: 10.3390/cancers15051610 (PMC10000504; doi:10.3390/cancers15051610)
Supplement: Supplementary file 1 [file cancers-15-01610-s001.zip › Sfigure.pdf]

Figure S1

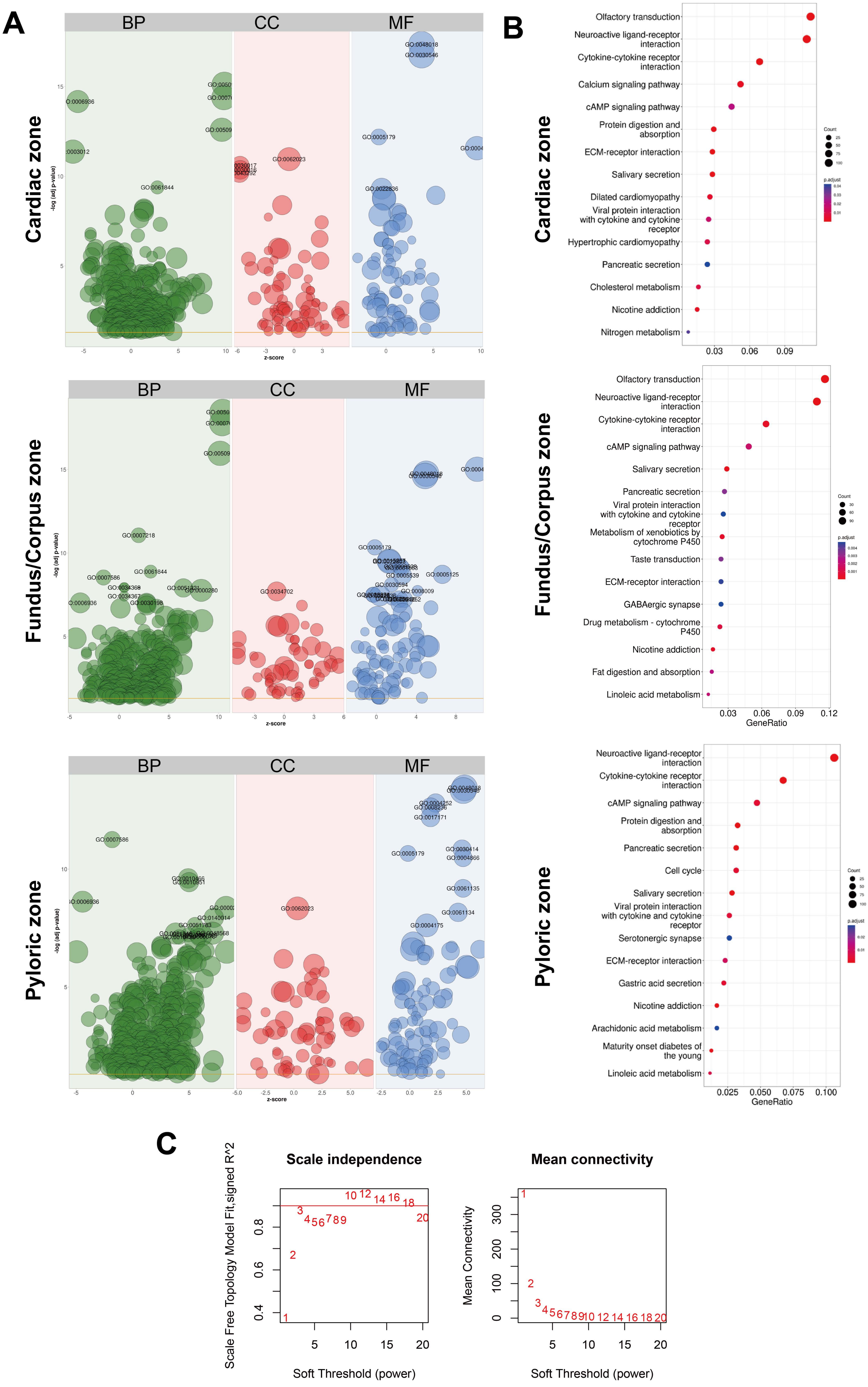

A
B
C
BP
CC
MF
Cardiac zone
Fundus/Corpus zone
Pyloric zone
-log (adj p-value)
z-score
Olfactory transduction
Neuroactive ligand-receptor interaction
Cytokine-cytokine receptor interaction
Calcium signaling pathway
cAMP signaling pathway
Protein digestion and absorption
ECM-receptor interaction
Salivary secretion
Dilated cardiomyopathy
Viral protein interaction with cytokine and cytokine receptor
Hypertrophic cardiomyopathy
Pancreatic secretion
Cholesterol metabolism
Nicotine addiction
Nitrogen metabolism
Metabolism of xenobiotics by cytochrome P450
Taste transduction
GABAergic synapse
Drug metabolism - cytochrome P450
Fat digestion and absorption
Linoleic acid metabolism
Cell cycle
Serotonergic synapse
Gastric acid secretion
Arachidonic acid metabolism
Maturity onset diabetes of the young
Count
p.adjust
GeneRatio
Scale independence
Mean connectivity
Scale Free Topology Model Fit,signed R^2
Mean Connectivity
Soft Threshold (power)

Figure S2

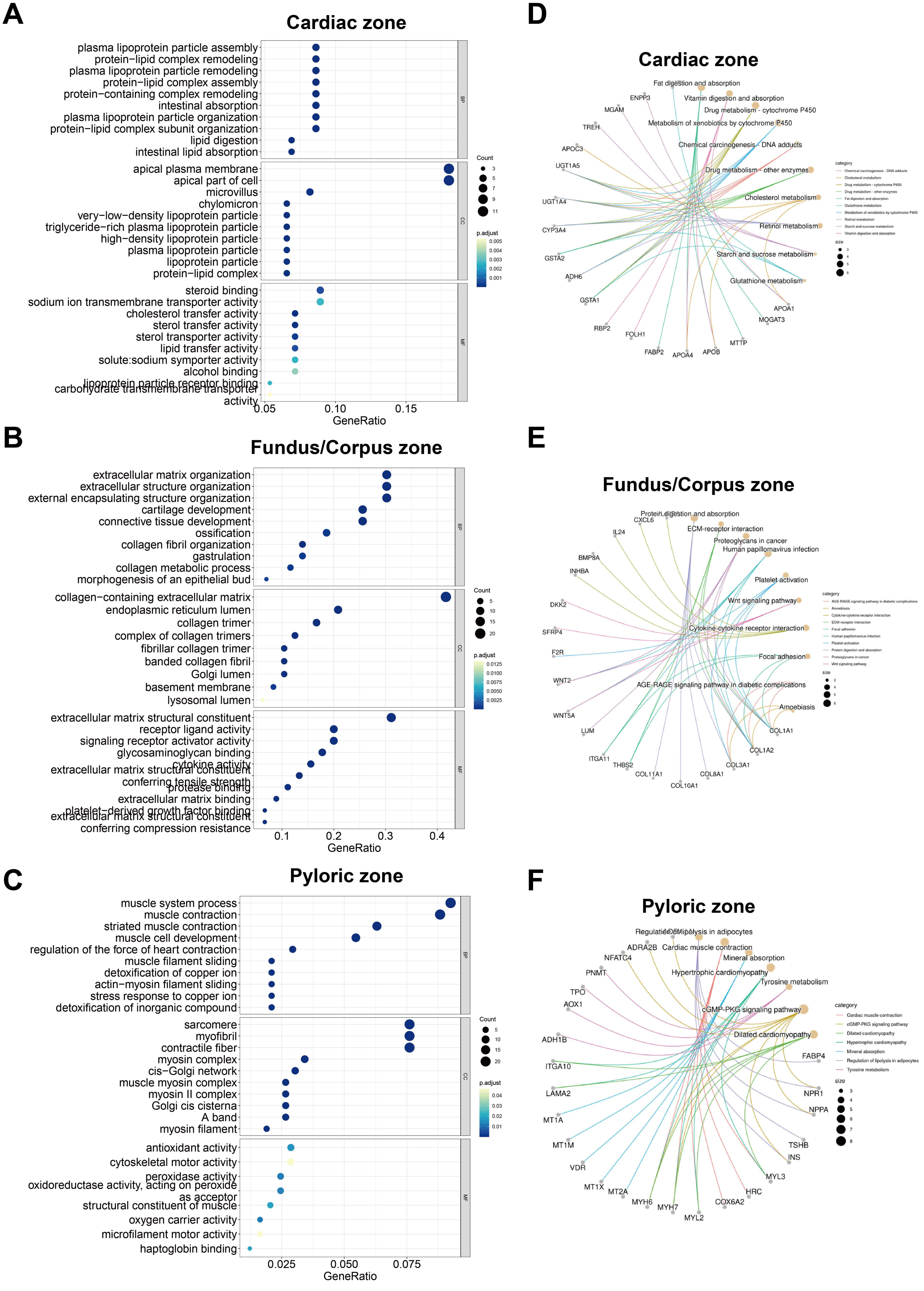

A
Cardiac zone
plasma lipoprotein particle assembly
protein−lipid complex remodeling
plasma lipoprotein particle remodeling
protein−lipid complex assembly
protein−containing complex remodeling
intestinal absorption
plasma lipoprotein particle organization
protein−lipid complex subunit organization
lipid digestion
intestinal lipid absorption
apical plasma membrane
apical part of cell
microvillus
chylomicron
very−low−density lipoprotein particle
triglyceride−rich plasma lipoprotein particle
high−density lipoprotein particle
plasma lipoprotein particle
lipoprotein particle
protein−lipid complex
steroid binding
sodium ion transmembrane transporter activity
cholesterol transfer activity
sterol transfer activity
sterol transporter activity
lipid transfer activity
solute:sodium symporter activity
alcohol binding
lipoprotein particle receptor binding
carbohydrate transmembrane transporter activity
BP
CC
MF
0.05
0.10
0.15
GeneRatio
Count
3
5
7
9
11
p.adjust
0.005
0.004
0.003
0.002
0.001
B
Fundus/Corpus zone
extracellular matrix organization
extracellular structure organization
external encapsulating structure organization
cartilage development
connective tissue development
ossification
collagen fibril organization
gastrulation
collagen metabolic process
morphogenesis of an epithelial bud
collagen−containing extracellular matrix
endoplasmic reticulum lumen
collagen trimer
complex of collagen trimers
fibrillar collagen trimer
banded collagen fibril
Golgi lumen
basement membrane
lysosomal lumen
extracellular matrix structural constituent
receptor ligand activity
signaling receptor activator activity
glycosaminoglycan binding
cytokine activity
extracellular matrix structural constituent conferring tensile strength
protease binding
extracellular matrix binding
platelet−derived growth factor binding
extracellular matrix structural constituent conferring compression resistance
BP
CC
MF
0.1
0.2
0.3
0.4
GeneRatio
Count
5
10
15
20
p.adjust
0.0125
0.0100
0.0075
0.0050
0.0025
C
Pyloric zone
muscle system process
muscle contraction
striated muscle contraction
muscle cell development
regulation of the force of heart contraction
muscle filament sliding
detoxification of copper ion
actin−myosin filament sliding
stress response to copper ion
detoxification of inorganic compound
sarcomere
myofibril
contractile fiber
myosin complex
cis−Golgi network
muscle myosin complex
myosin II complex
Golgi cis cisterna
A band
myosin filament
antioxidant activity
cytoskeletal motor activity
peroxidase activity
oxidoreductase activity, acting on peroxide as acceptor
structural constituent of muscle
oxygen carrier activity
microfilament motor activity
haptoglobin binding
BP
CC
MF
0.025
0.050
0.075
GeneRatio
Count
5
10
15
20
p.adjust
0.04
0.03
0.02
0.01
D
Cardiac zone
Fat digestion and absorption
Vitamin digestion and absorption
Drug metabolism - cytochrome P450
Metabolism of xenobiotics by cytochrome P450
Chemical carcinogenesis - DNA adducts
Drug metabolism - other enzymes
Cholesterol metabolism
Retinol metabolism
Starch and sucrose metabolism
Glutathione metabolism
ENPP3
MGAM
TREH
APOC3
UGT1A5
UGT1A4
CYP3A4
GSTA2
ADH6
GSTA1
RBP2
FOLH1
FABP2
APOA4
APOB
MTTP
MOGAT3
APOA1
category
Chemical carcinogenesis - DNA adducts
Cholesterol metabolism
Drug metabolism - cytochrome P450
Drug metabolism - other enzymes
Fat digestion and absorption
Glutathione metabolism
Metabolism of xenobiotics by cytochrome P450
Retinol metabolism
Starch and sucrose metabolism
Vitamin digestion and absorption
size
3
4
5
6
E
Fundus/Corpus zone
Protein digestion and absorption
ECM-receptor interaction
Proteoglycans in cancer
Human papillomavirus infection
Platelet activation
Wnt signaling pathway
Cytokine-cytokine receptor interaction
Focal adhesion
AGE-RAGE signaling pathway in diabetic complications
Amoebiasis
CXCL6
IL24
BMP8A
INHBA
DKK2
SFRP4
F2R
WNT2
WNT5A
LUM
ITGA11
THBS2
COL11A1
COL10A1
COL8A1
COL3A1
COL1A2
COL1A1
category
AGE-RAGE signaling pathway in diabetic complications
Amoebiasis
Cytokine-cytokine receptor interaction
ECM-receptor interaction
Focal adhesion
Human papillomavirus infection
Platelet activation
Protein digestion and absorption
Proteoglycans in cancer
Wnt signaling pathway
size
3
4
5
6
F
Pyloric zone
Regulation of lipolysis in adipocytes
Cardiac muscle contraction
Mineral absorption
Hypertrophic cardiomyopathy
Tyrosine metabolism
cGMP-PKG signaling pathway
Dilated cardiomyopathy
ADRA2B
NFATC4
PNMT
TPO
AOX1
ADH1B
ITGA10
LAMA2
MT1A
MT1M
VDR
MT1X
MT2A
MYH6
MYH7
MYL2
COX6A2
HRC
MYL3
INS
TSHB
NPPA
NPR1
FABP4
category
Cardiac muscle contraction
cGMP-PKG signaling pathway
Dilated cardiomyopathy
Hypertrophic cardiomyopathy
Mineral absorption
Regulation of lipolysis in adipocytes
Tyrosine metabolism
size
3
4
5
6
7
8

Figure S3

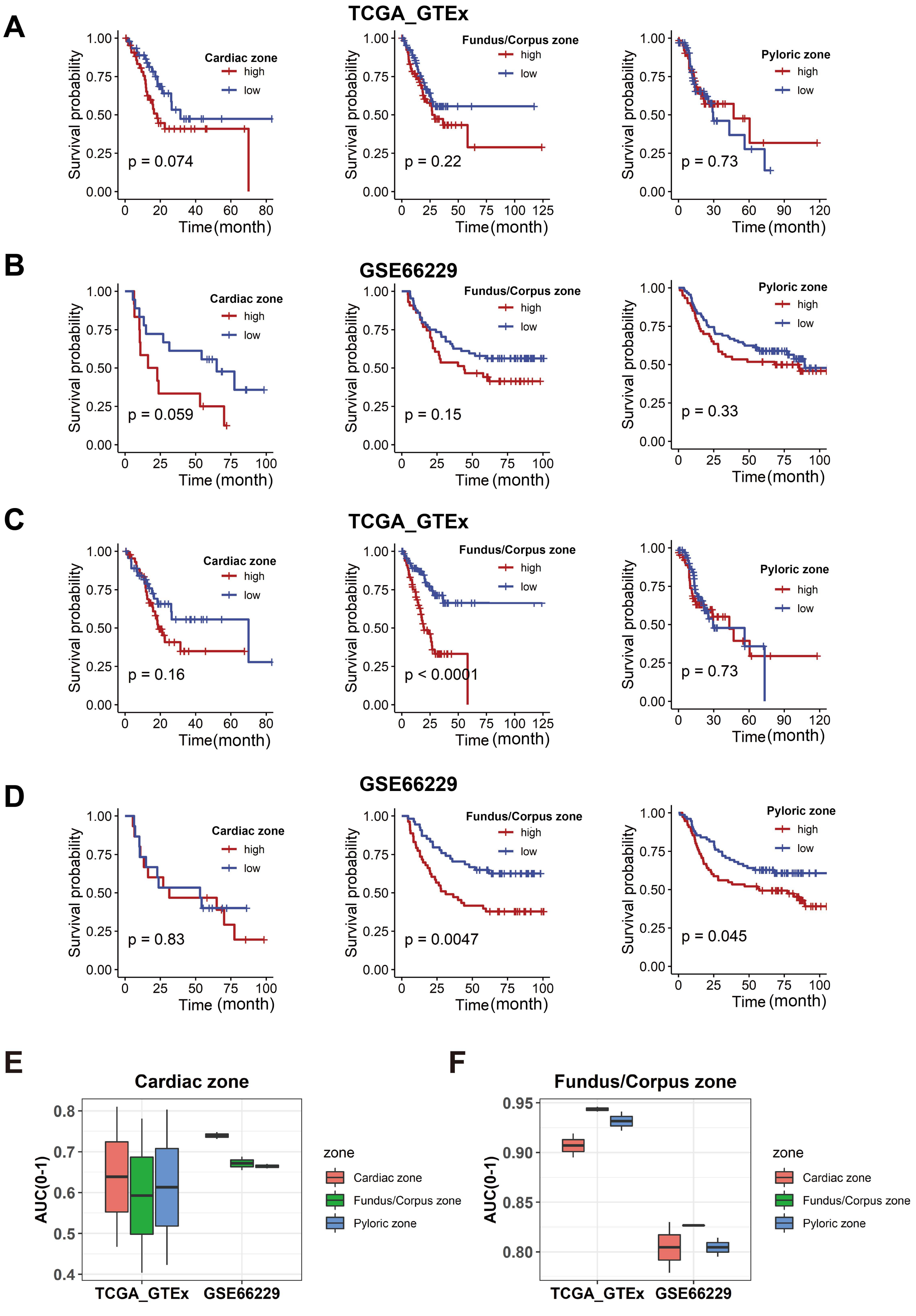

A
TCGA_GTEx
Cardiac zone
high
low
p = 0.074
Fundus/Corpus zone
p = 0.22
Pyloric zone
p = 0.73
Survival probability
Time(month)
B
GSE66229
p = 0.059
p = 0.15
p = 0.33
C
TCGA_GTEx
p = 0.16
p < 0.0001
p = 0.73
D
GSE66229
p = 0.83
p = 0.0047
p = 0.045
E
Cardiac zone
AUC(0-1)
TCGA_GTEx
GSE66229
zone
Cardiac zone
Fundus/Corpus zone
Pyloric zone
F
Fundus/Corpus zone

Figure S4

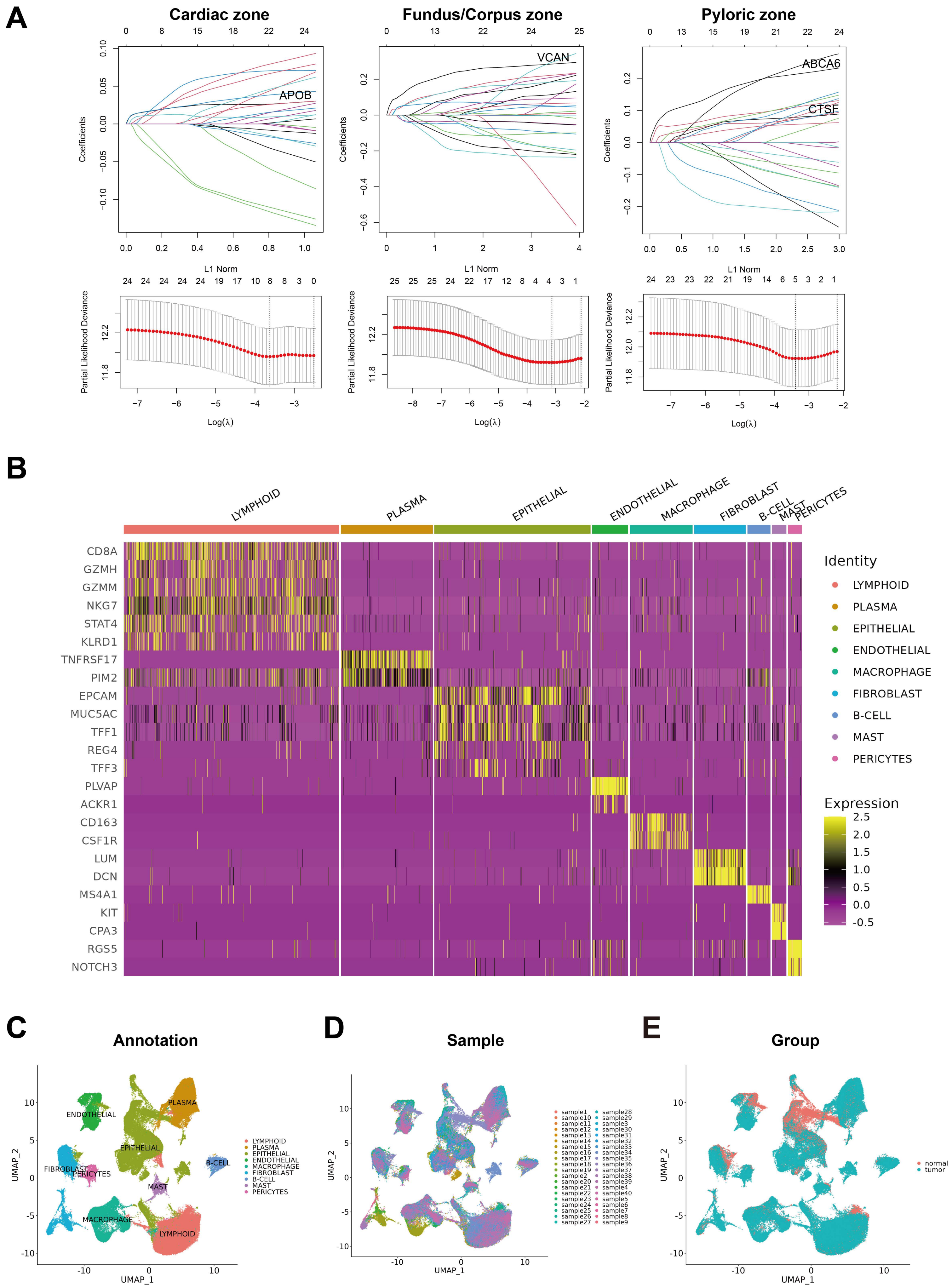
A
Cardiac zone
Fundus/Corpus zone
Pyloric zone
APOB
VCAN
ABCA6
CTSF
Coefficients
L1 Norm
Partial Likelihood Deviance
Log(λ)
B
LYMPHOID
PLASMA
EPITHELIAL
ENDOTHELIAL
MACROPHAGE
FIBROBLAST
B-CELL
MAST
PERICYTES
CD8A
GZMH
GZMM
NKG7
STAT4
KLRD1
TNFRSF17
PIM2
EPCAM
MUC5AC
TFF1
REG4
TFF3
PLVAP
ACKR1
CD163
CSF1R
LUM
DCN
MS4A1
KIT
CPA3
RGS5
NOTCH3
Identity
Expression
C
Annotation
D
Sample
E
Group
UMAP_1
UMAP_2
normal
tumor

Figure S5

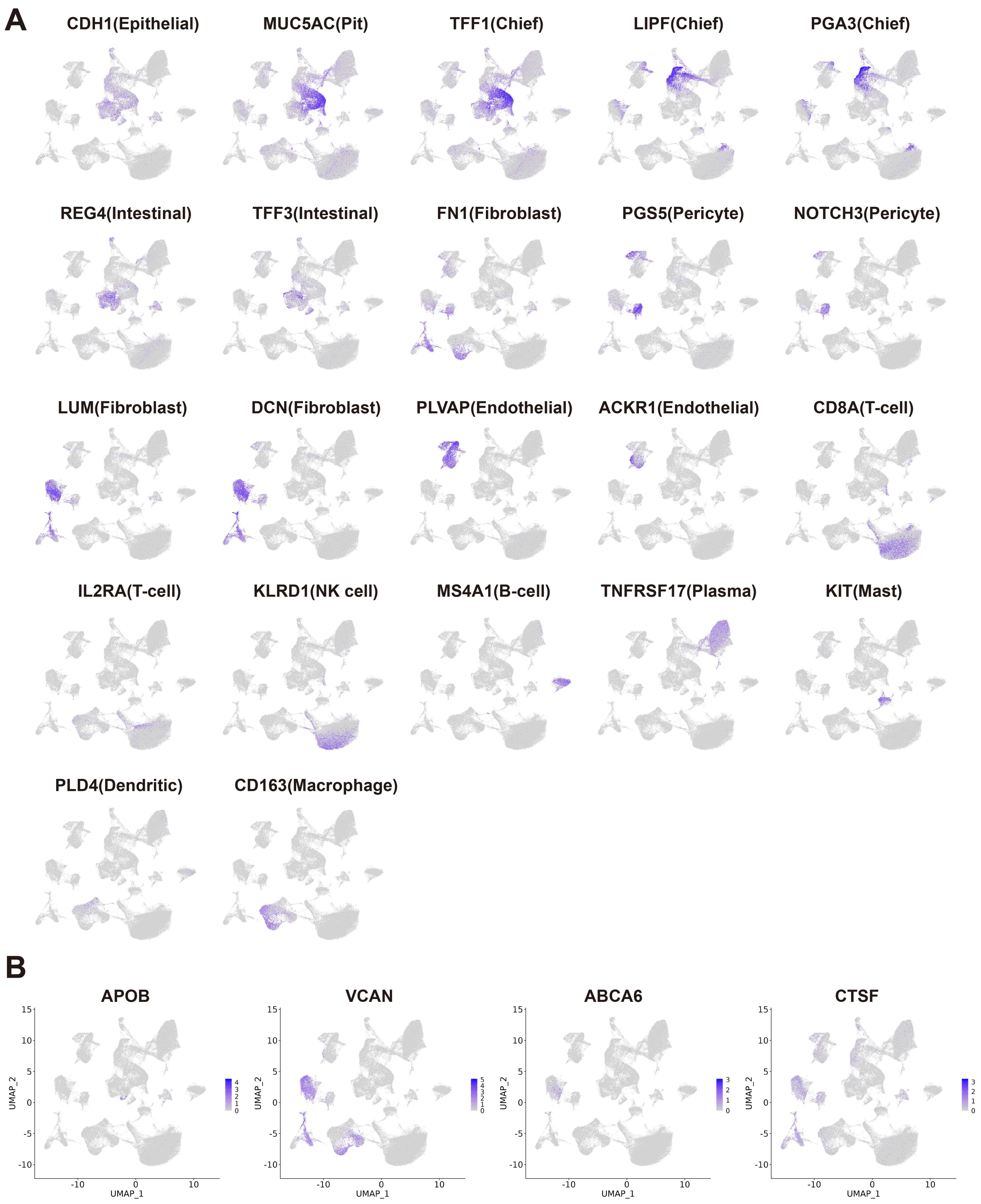
A
CDH1(Epithelial)
MUC5AC(Pit)
TFF1(Chief)
LIPF(Chief)
PGA3(Chief)
REG4(Intestinal)
TFF3(Intestinal)
FN1(Fibroblast)
PGS5(Pericyte)
NOTCH3(Pericyte)
LUM(Fibroblast)
DCN(Fibroblast)
PLVAP(Endothelial)
ACKR1(Endothelial)
CD8A(T-cell)
IL2RA(T-cell)
KLRD1(NK cell)
MS4A1(B-cell)
TNFRSF17(Plasma)
KIT(Mast)
PLD4(Dendritic)
CD163(Macrophage)
B
APOB
VCAN
ABCA6
CTSF
UMAP_1
UMAP_2

Figure S6

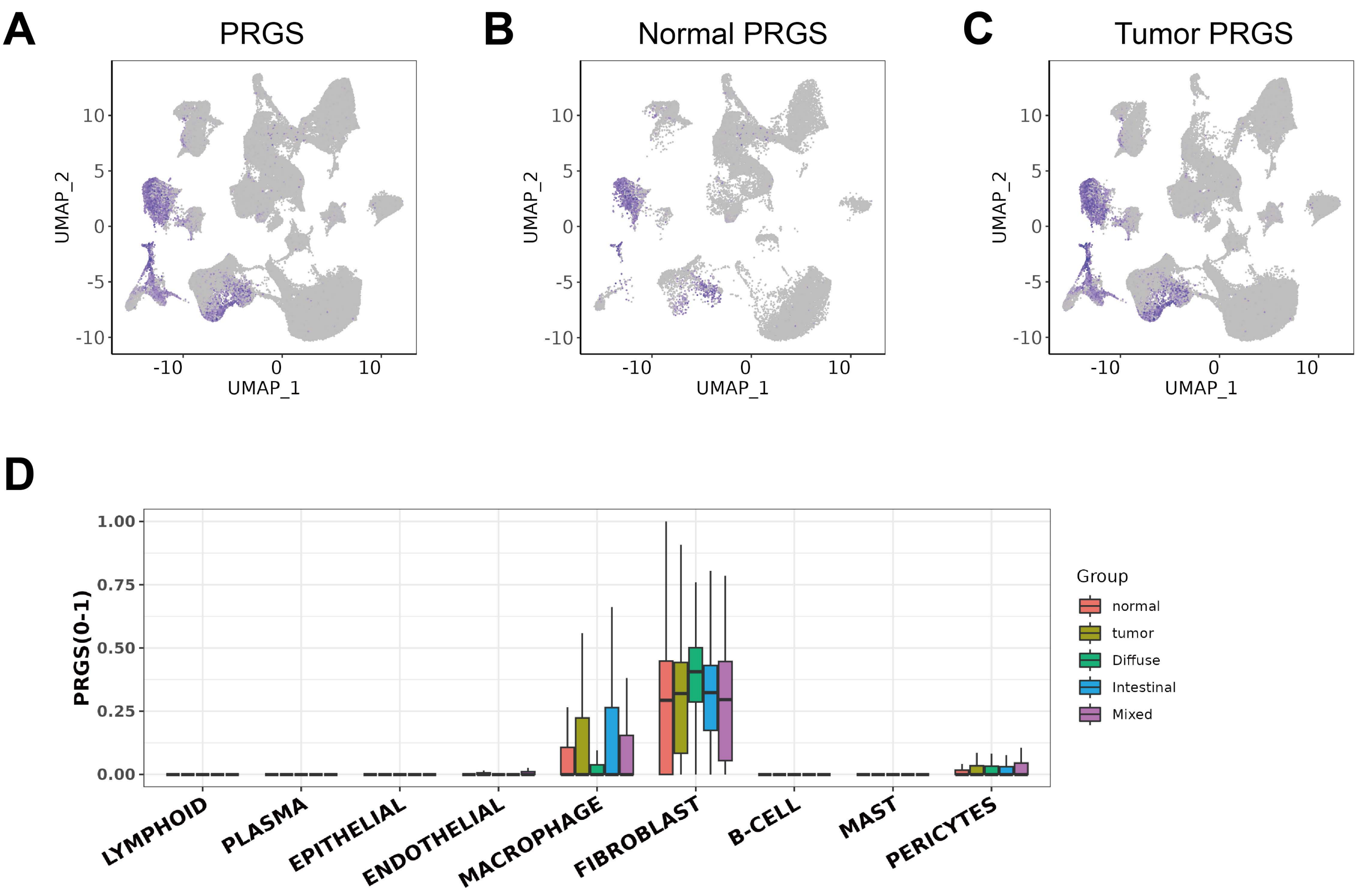
A
PRGS
B
Normal PRGS
C
Tumor PRGS
UMAP_1
UMAP_2
D
PRGS(0-1)
Group
normal
tumor
Diffuse
Intestinal
Mixed
LYMPHOID
PLASMA
EPITHELIAL
ENDOTHELIAL
MACROPHAGE
FIBROBLAST
B-CELL
MAST
PERICYTES

Figure S7

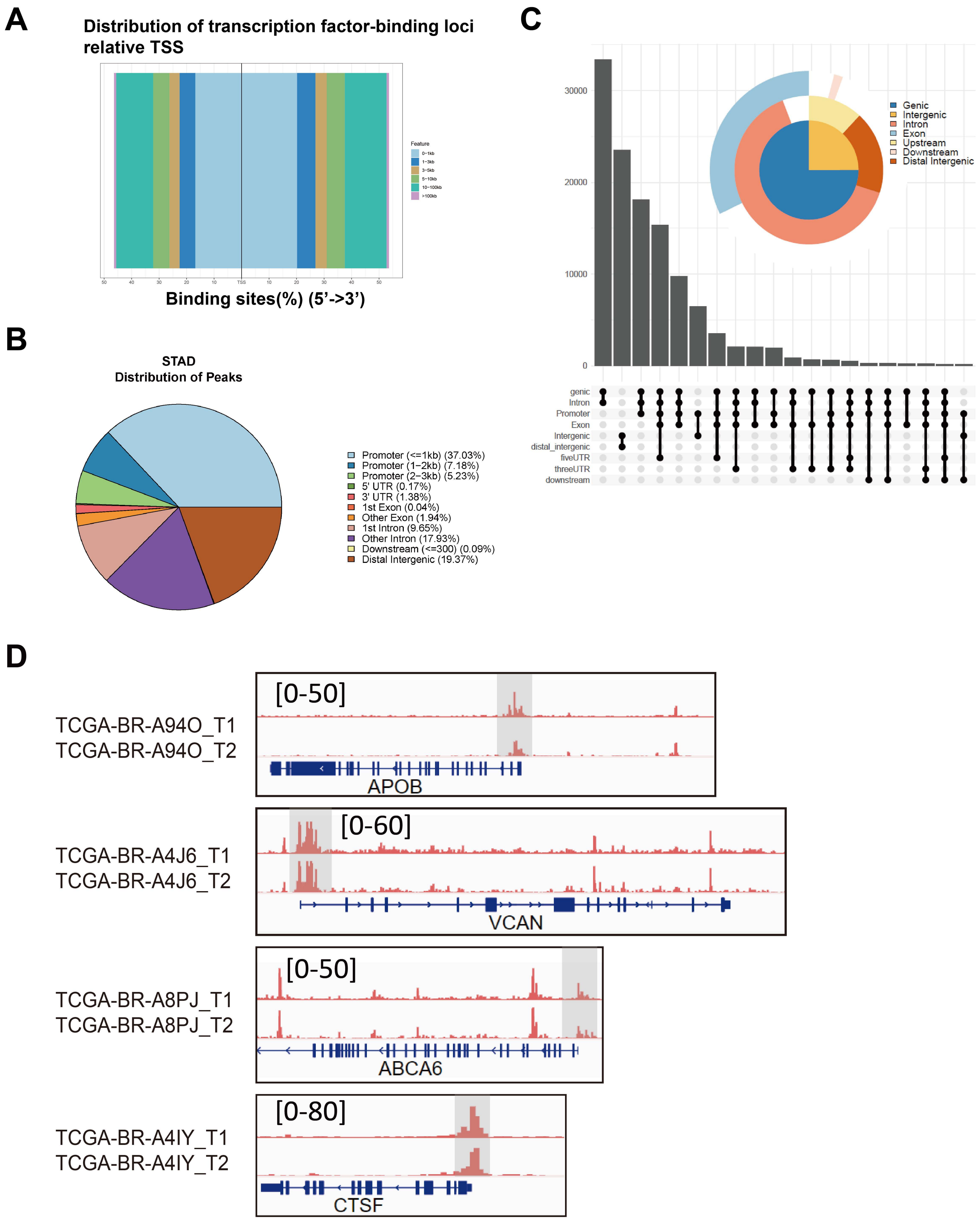
A
Distribution of transcription factor-binding loci relative TSS
Feature
0~1kb
1~3kb
3~5kb
5~10kb
10~100kb
>100kb
Binding sites(%) (5'->3')
B
STAD
Distribution of Peaks
Promoter (<=1kb) (37.03%)
Promoter (1−2kb) (7.18%)
Promoter (2−3kb) (5.23%)
5' UTR (0.17%)
3' UTR (1.38%)
1st Exon (0.04%)
Other Exon (1.94%)
1st Intron (9.65%)
Other Intron (17.93%)
Downstream (<=300) (0.09%)
Distal Intergenic (19.37%)
C
Genic
Intergenic
Intron
Exon
Upstream
Downstream
Distal Intergenic
genic
Intron
Promoter
Exon
Intergenic
distal_intergenic
fiveUTR
threeUTR
downstream
D
TCGA-BR-A94O_T1
TCGA-BR-A94O_T2
[0-50]
APOB
TCGA-BR-A4J6_T1
TCGA-BR-A4J6_T2
[0-60]
VCAN
TCGA-BR-A8PJ_T1
TCGA-BR-A8PJ_T2
[0-50]
ABCA6
TCGA-BR-A4IY_T1
TCGA-BR-A4IY_T2
[0-80]
CTSF

Figure S8

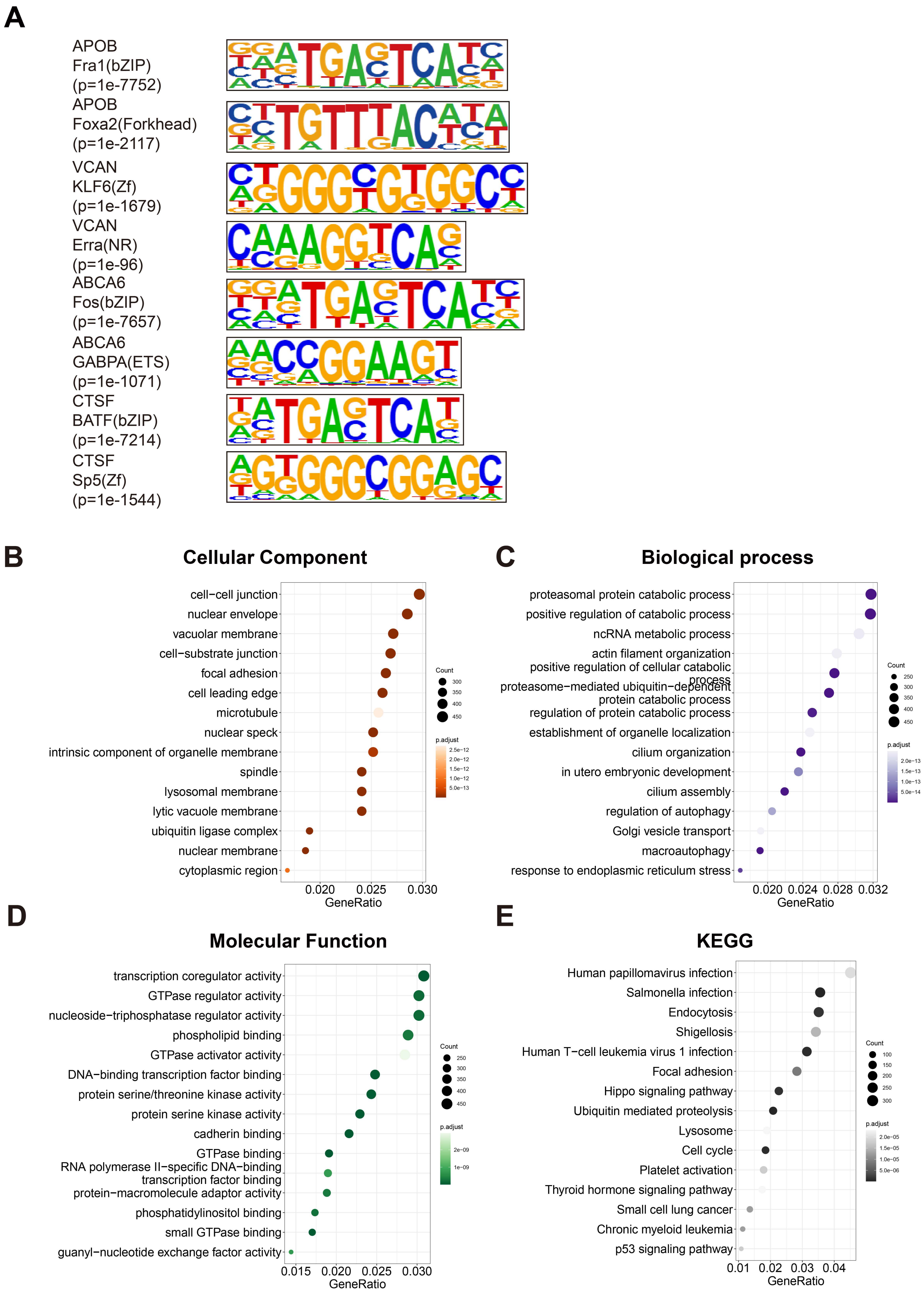
A
APOB
Fra1(bZIP)
(p=1e-7752)
APOB
Foxa2(Forkhead)
(p=1e-2117)
VCAN
KLF6(Zf)
(p=1e-1679)
VCAN
Erra(NR)
(p=1e-96)
ABCA6
Fos(bZIP)
(p=1e-7657)
ABCA6
GABPA(ETS)
(p=1e-1071)
CTSF
BATF(bZIP)
(p=1e-7214)
CTSF
Sp5(Zf)
(p=1e-1544)
B
Cellular Component
cell−cell junction
nuclear envelope
vacuolar membrane
cell−substrate junction
focal adhesion
cell leading edge
microtubule
nuclear speck
intrinsic component of organelle membrane
spindle
lysosomal membrane
lytic vacuole membrane
ubiquitin ligase complex
nuclear membrane
cytoplasmic region
0.020
0.025
0.030
GeneRatio
Count
300
350
400
450
p.adjust
2.5e−12
2.0e−12
1.5e−12
1.0e−12
5.0e−13
C
Biological process
proteasomal protein catabolic process
positive regulation of catabolic process
ncRNA metabolic process
actin filament organization
positive regulation of cellular catabolic process
proteasome−mediated ubiquitin−dependent protein catabolic process
regulation of protein catabolic process
establishment of organelle localization
cilium organization
in utero embryonic development
cilium assembly
regulation of autophagy
Golgi vesicle transport
macroautophagy
response to endoplasmic reticulum stress
0.020
0.024
0.028
0.032
GeneRatio
Count
250
300
350
400
450
p.adjust
2.0e−13
1.5e−13
1.0e−13
5.0e−14
D
Molecular Function
transcription coregulator activity
GTPase regulator activity
nucleoside−triphosphatase regulator activity
phospholipid binding
GTPase activator activity
DNA−binding transcription factor binding
protein serine/threonine kinase activity
protein serine kinase activity
cadherin binding
GTPase binding
RNA polymerase II−specific DNA−binding transcription factor binding
protein−macromolecule adaptor activity
phosphatidylinositol binding
small GTPase binding
guanyl−nucleotide exchange factor activity
0.015
0.020
0.025
0.030
GeneRatio
Count
250
300
350
400
450
p.adjust
2e−09
1e−09
E
KEGG
Human papillomavirus infection
Salmonella infection
Endocytosis
Shigellosis
Human T−cell leukemia virus 1 infection
Focal adhesion
Hippo signaling pathway
Ubiquitin mediated proteolysis
Lysosome
Cell cycle
Platelet activation
Thyroid hormone signaling pathway
Small cell lung cancer
Chronic myeloid leukemia
p53 signaling pathway
0.01
0.02
0.03
0.04
GeneRatio
Count
100
150
200
250
300
p.adjust
2.0e−05
1.5e−05
1.0e−05
5.0e−06

Figure S9

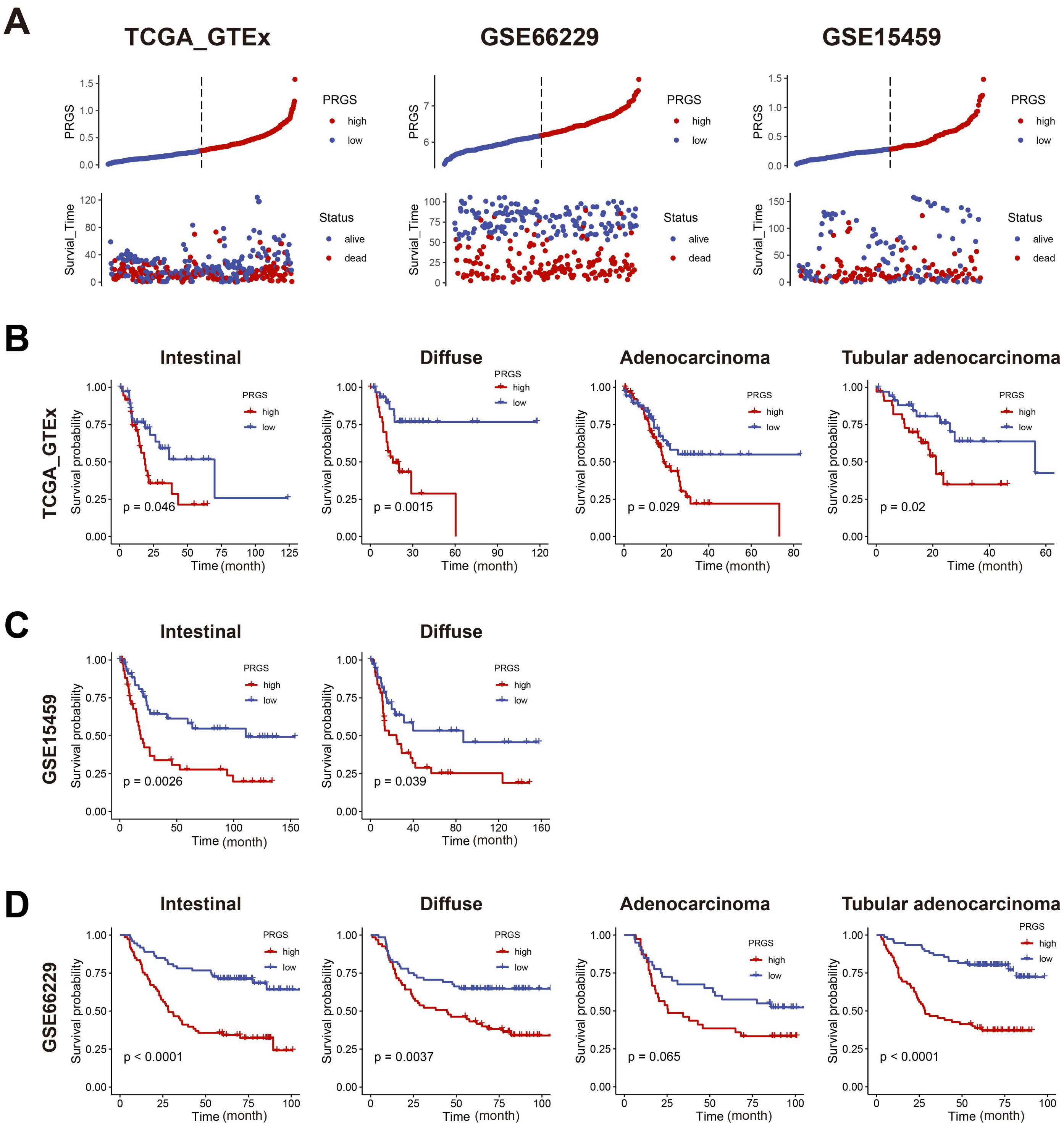
A
TCGA_GTEx
GSE66229
GSE15459
PRGS
high
low
Survial_Time
Status
alive
dead
B
Intestinal
Diffuse
Adenocarcinoma
Tubular adenocarcinoma
TCGA_GTEx
Survival probability
p = 0.046
p = 0.0015
p = 0.029
p = 0.02
Time (month)
C
GSE15459
p = 0.0026
p = 0.039
D
GSE66229
p < 0.0001
p = 0.0037
p = 0.065
p < 0.0001

Figure S10

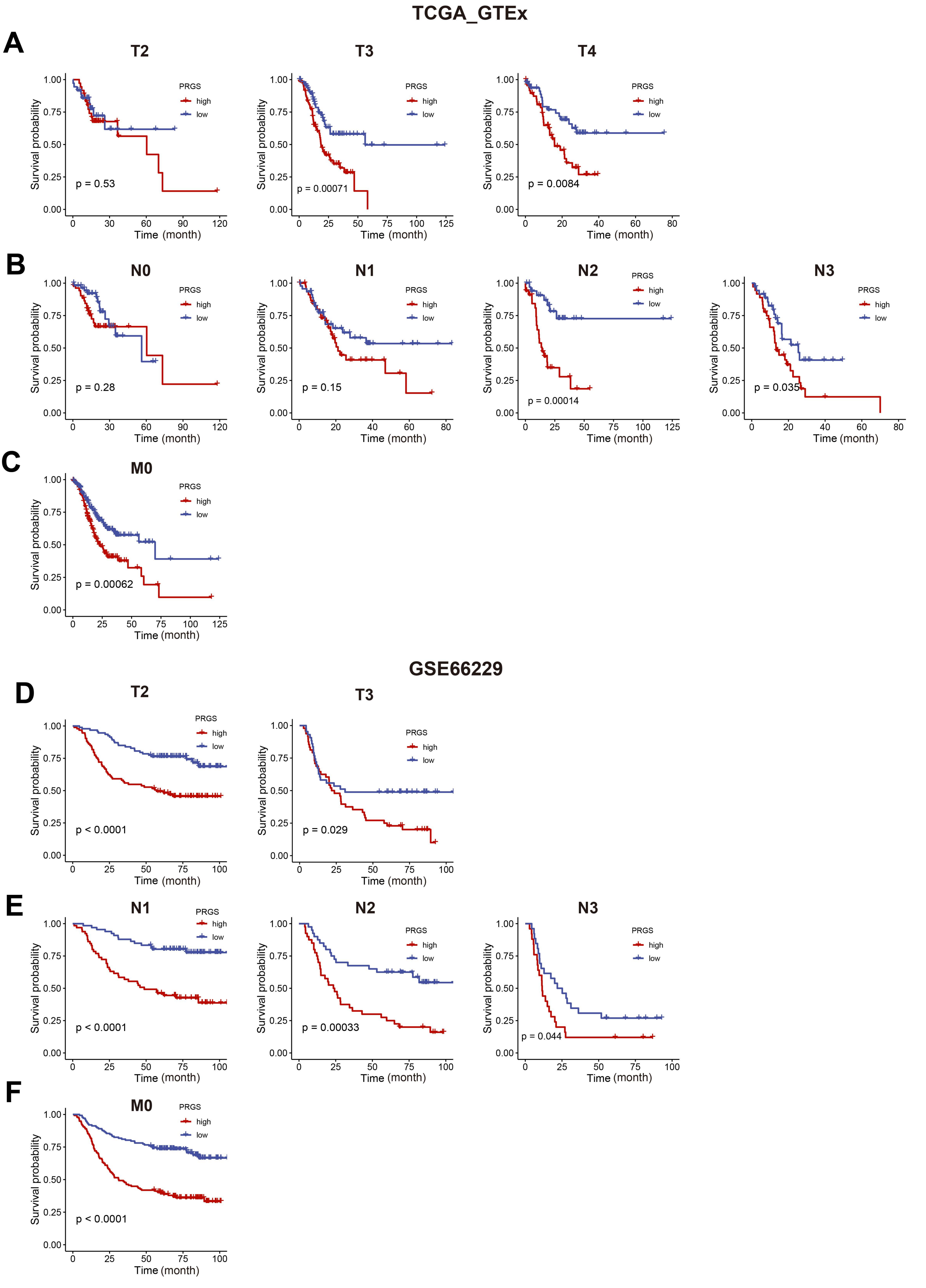

TCGA_GTEx
A
T2
PRGS
high
low
p = 0.53
T3
p = 0.00071
T4
p = 0.0084
B
N0
p = 0.28
N1
p = 0.15
N2
p = 0.00014
N3
p = 0.035
C
M0
p = 0.00062
Survival probability
Time (month)
GSE66229
D
T2
p < 0.0001
T3
p = 0.029
E
N1
p < 0.0001
N2
p = 0.00033
N3
p = 0.044
F
M0
p < 0.0001

Figure S11

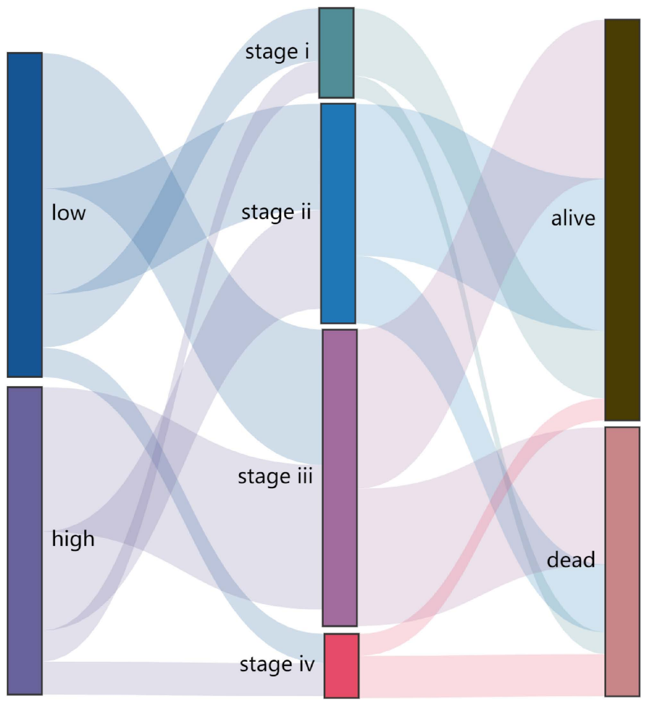

Figure S12

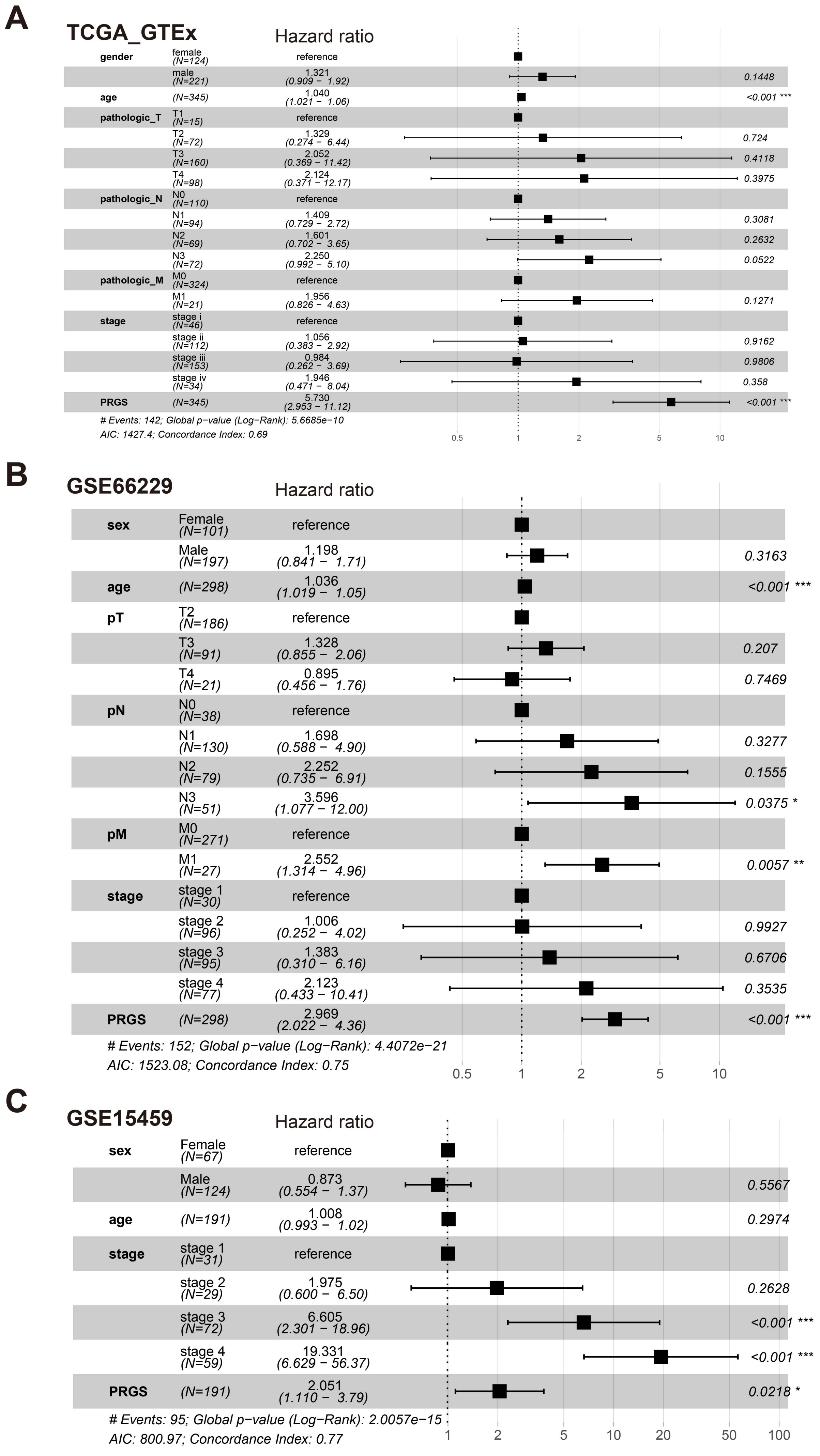
A
TCGA_GTEx
Hazard ratio
gender
female (N=124) reference
male (N=221) 1.321 (0.909 − 1.92) 0.1448
age (N=345) 1.040 (1.021 − 1.06) <0.001 ***
pathologic_T
T1 (N=15) reference
T2 (N=72) 1.329 (0.274 − 6.44) 0.724
T3 (N=160) 2.052 (0.369 − 11.42) 0.4118
T4 (N=98) 2.124 (0.371 − 12.17) 0.3975
pathologic_N
N0 (N=110) reference
N1 (N=94) 1.409 (0.729 − 2.72) 0.3081
N2 (N=69) 1.601 (0.702 − 3.65) 0.2632
N3 (N=72) 2.250 (0.992 − 5.10) 0.0522
pathologic_M
M0 (N=324) reference
M1 (N=21) 1.956 (0.826 − 4.63) 0.1271
stage
stage i (N=46) reference
stage ii (N=112) 1.056 (0.383 − 2.92) 0.9162
stage iii (N=153) 0.984 (0.262 − 3.69) 0.9806
stage iv (N=34) 1.946 (0.471 − 8.04) 0.358
PRGS (N=345) 5.730 (2.953 − 11.12) <0.001 ***
Events: 142; Global p−value (Log−Rank): 5.6685e−10
AIC: 1427.4; Concordance Index: 0.69
0.5 1 2 5 10
B
GSE66229
Hazard ratio
sex
Female (N=101) reference
Male (N=197) 1.198 (0.841 − 1.71) 0.3163
age (N=298) 1.036 (1.019 − 1.05) <0.001 ***
pT
T2 (N=186) reference
T3 (N=91) 1.328 (0.855 − 2.06) 0.207
T4 (N=21) 0.895 (0.456 − 1.76) 0.7469
pN
N0 (N=38) reference
N1 (N=130) 1.698 (0.588 − 4.90) 0.3277
N2 (N=79) 2.252 (0.735 − 6.91) 0.1555
N3 (N=51) 3.596 (1.077 − 12.00) 0.0375 *
pM
M0 (N=271) reference
M1 (N=27) 2.552 (1.314 − 4.96) 0.0057 **
stage
stage 1 (N=30) reference
stage 2 (N=96) 1.006 (0.252 − 4.02) 0.9927
stage 3 (N=95) 1.383 (0.310 − 6.16) 0.6706
stage 4 (N=77) 2.123 (0.433 − 10.41) 0.3535
PRGS (N=298) 2.969 (2.022 − 4.36) <0.001 ***
Events: 152; Global p−value (Log−Rank): 4.4072e−21
AIC: 1523.08; Concordance Index: 0.75
0.5 1 2 5 10
C
GSE15459
Hazard ratio
sex
Female (N=67) reference
Male (N=124) 0.873 (0.554 − 1.37) 0.5567
age (N=191) 1.008 (0.993 − 1.02) 0.2974
stage
stage 1 (N=31) reference
stage 2 (N=29) 1.975 (0.600 − 6.50) 0.2628
stage 3 (N=72) 6.605 (2.301 − 18.96) <0.001 ***
stage 4 (N=59) 19.331 (6.629 − 56.37) <0.001 ***
PRGS (N=191) 2.051 (1.110 − 3.79) 0.0218 *
Events: 95; Global p−value (Log−Rank): 2.0057e−15
AIC: 800.97; Concordance Index: 0.77
1 2 5 10 20 50 100

Figure S13

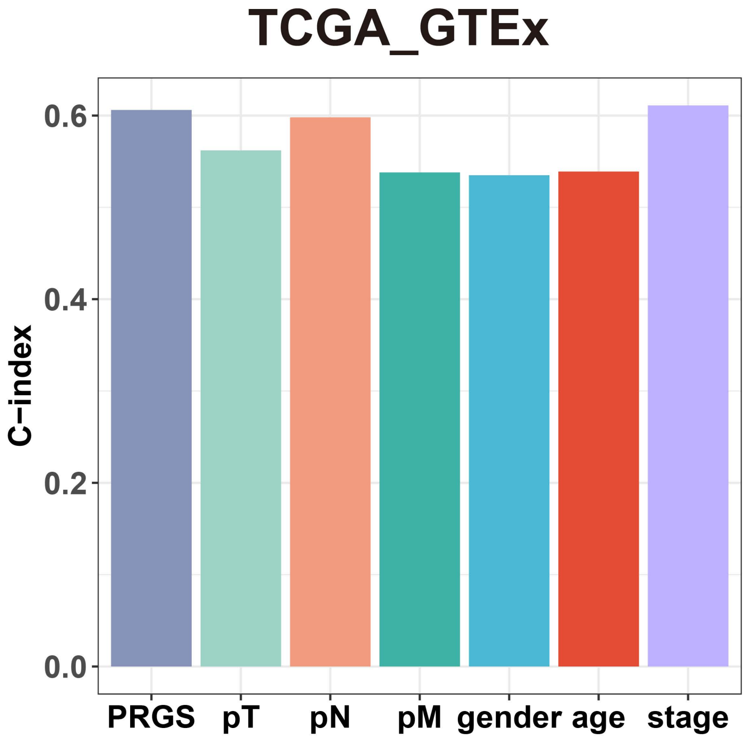

Figure S14

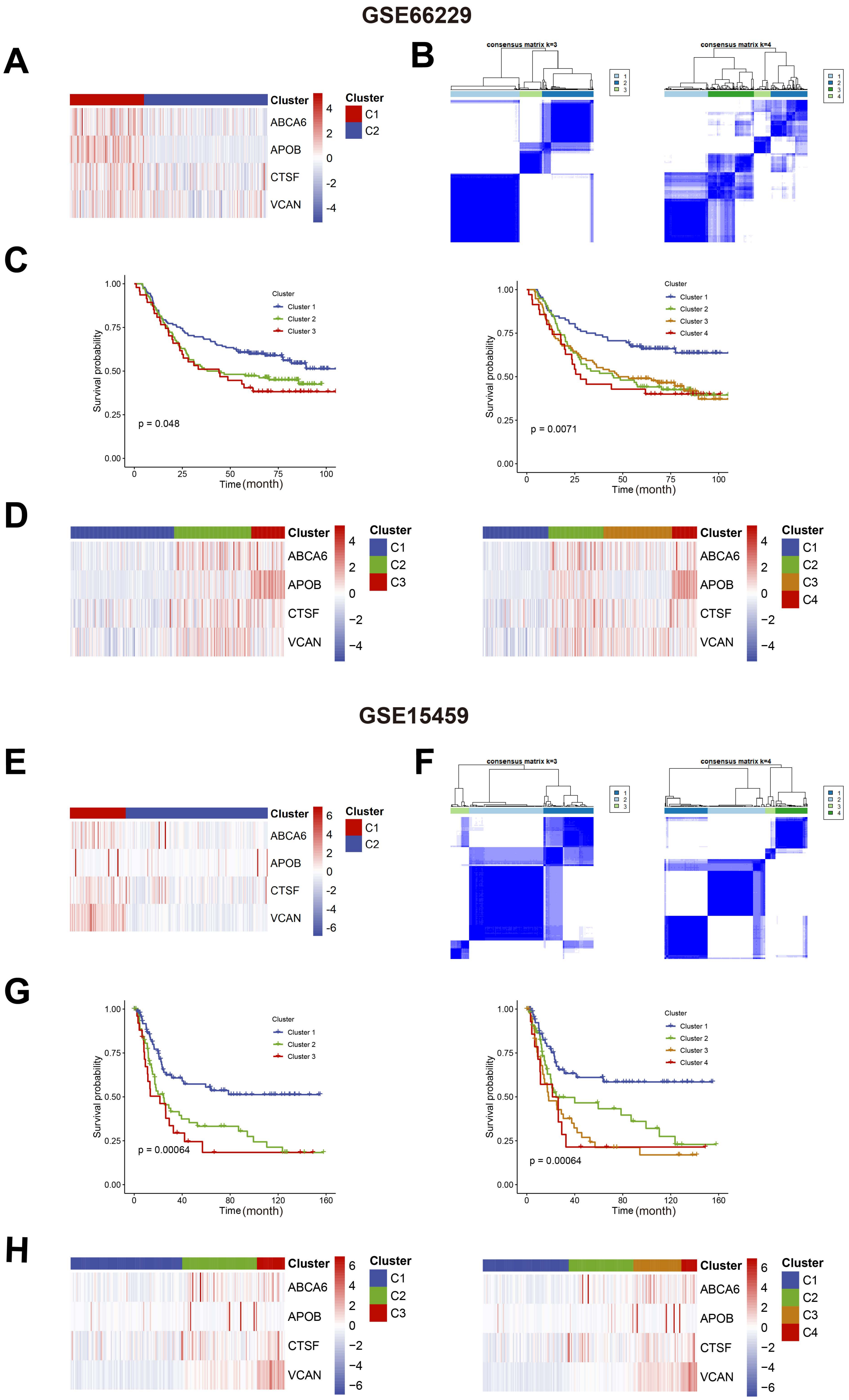
GSE66229
A
B
C
D
Cluster
ABCA6
APOB
CTSF
VCAN
C1
C2
C3
C4
consensus matrix k=3
consensus matrix k=4
Cluster 1
Cluster 2
Cluster 3
Cluster 4
Survival probability
Time (month)
p = 0.048
p = 0.0071
GSE15459
E
F
G
H
p = 0.00064
p = 0.00064

Figure S15

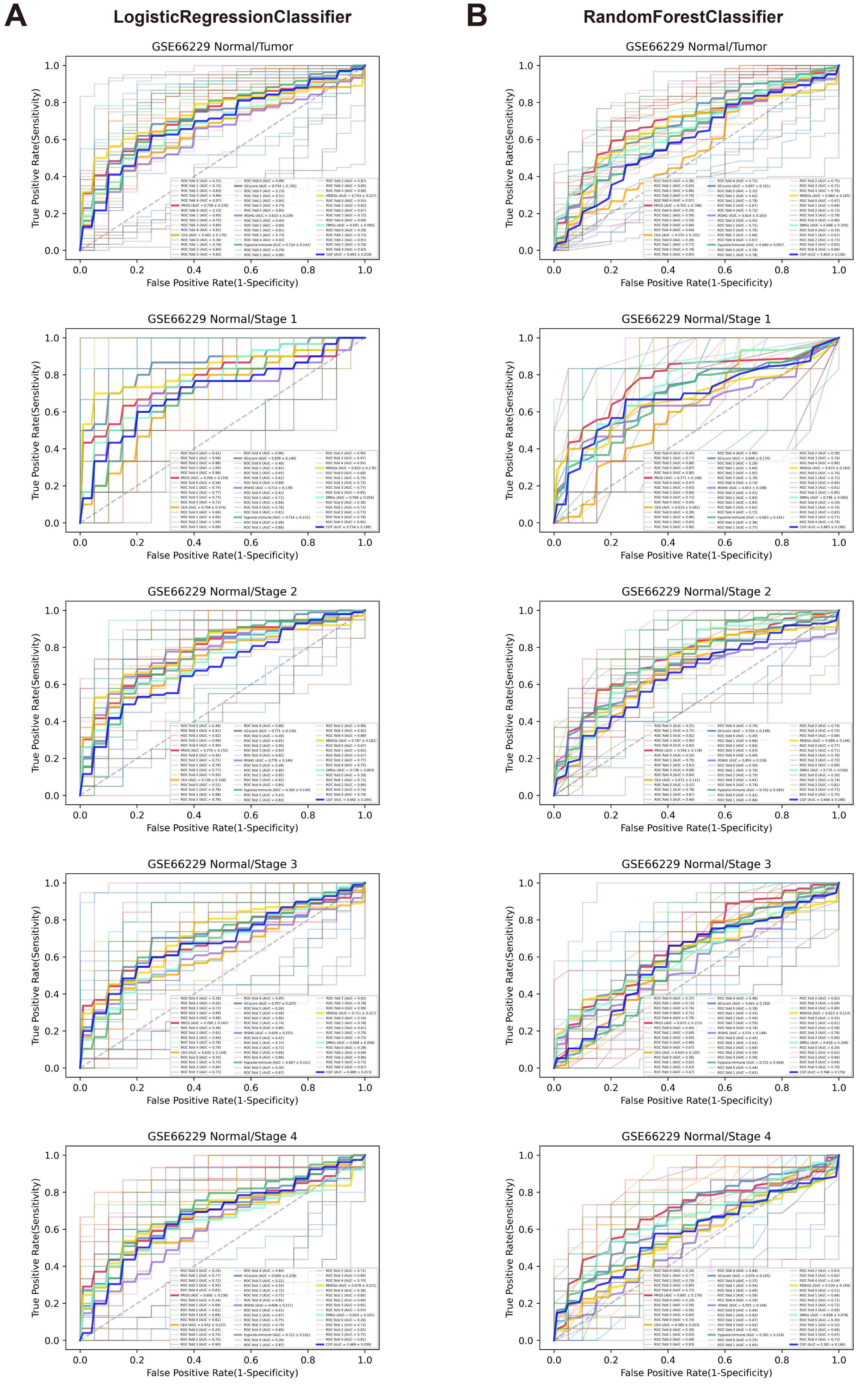
A
LogisticRegressionClassifier
B
RandomForestClassifier
GSE66229 Normal/Tumor
GSE66229 Normal/Stage 1
GSE66229 Normal/Stage 2
GSE66229 Normal/Stage 3
GSE66229 Normal/Stage 4
True Positive Rate(Sensitivity)
False Positive Rate(1-Specificity)

Figure S16

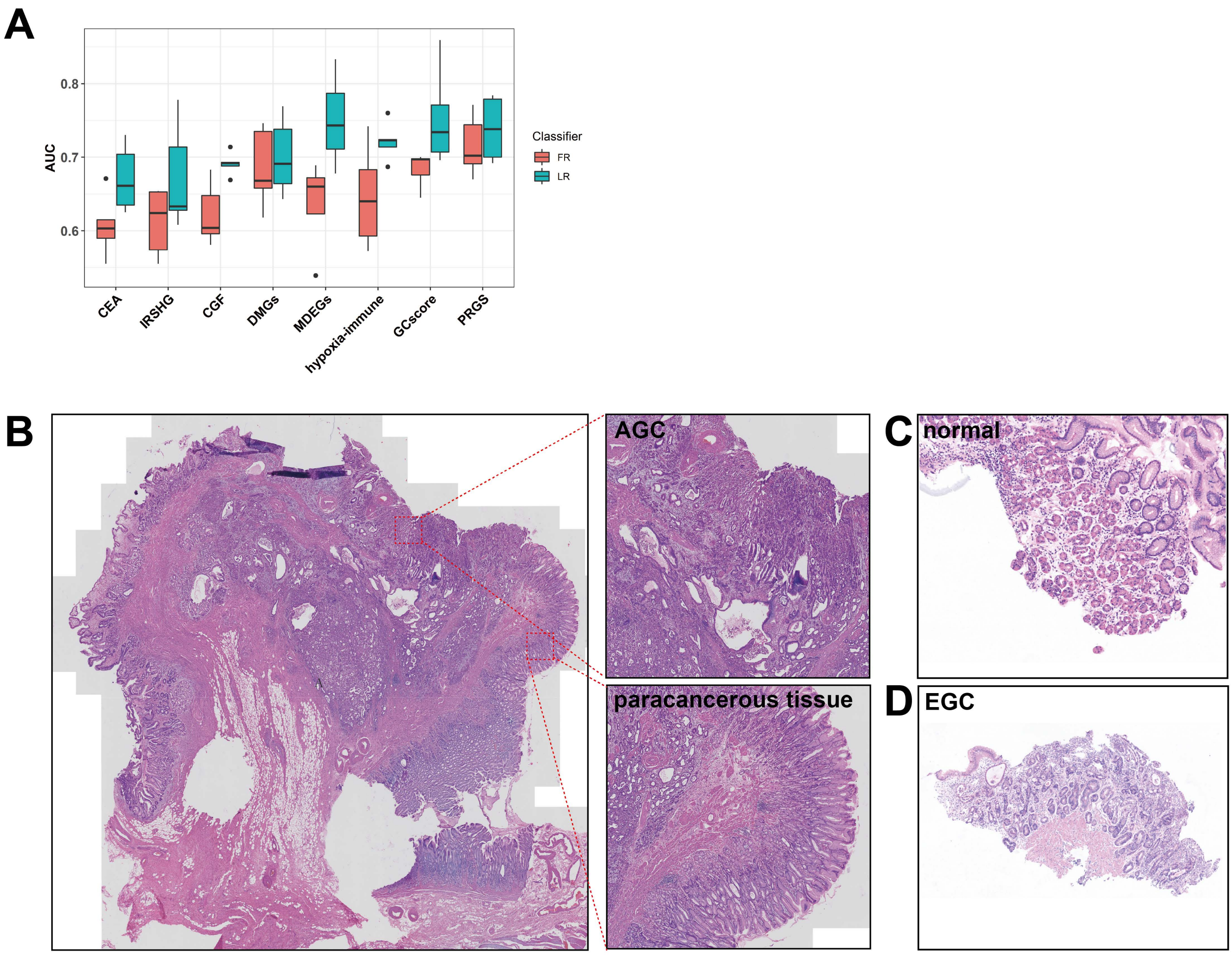

A
AUC
0.8
0.7
0.6
Classifier
FR
LR
CEA
IRSHG
CGF
DMGs
MDEGs
hypoxia-immune
GCscore
PRGS
B
AGC
paracancerous tissue
C
normal
D
EGC

Figure S17

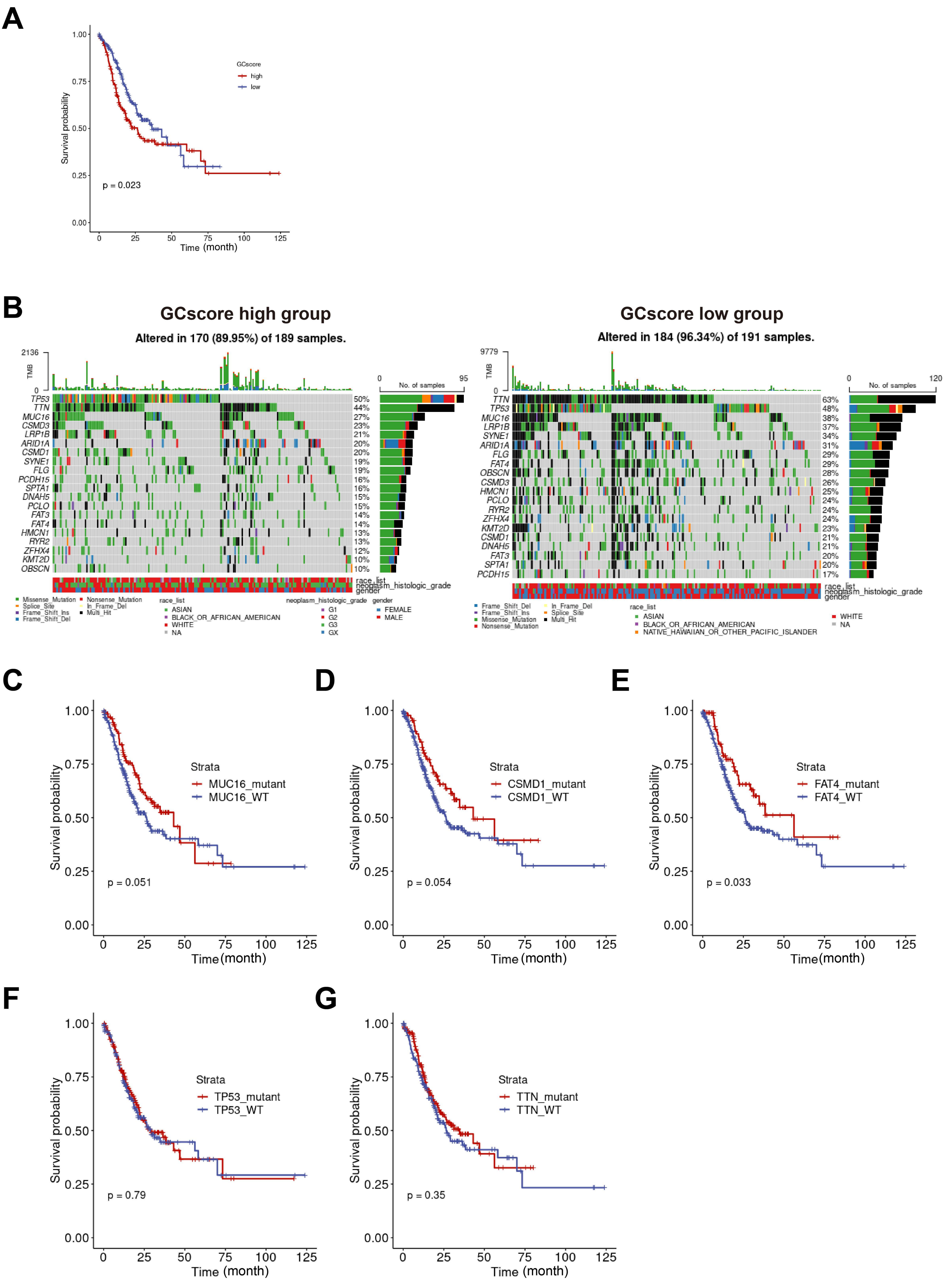
A
Survival probability
Time (month)
GCscore
high
low
p = 0.023
B
GCscore high group
Altered in 170 (89.95%) of 189 samples.
GCscore low group
Altered in 184 (96.34%) of 191 samples.
TMB
No. of samples
race_list
neoplasm_histologic_grade
gender
Missense_Mutation
Nonsense_Mutation
Splice_Site
In_Frame_Del
Frame_Shift_Ins
Frame_Shift_Del
Multi_Hit
ASIAN
BLACK_OR_AFRICAN_AMERICAN
NATIVE_HAWAIIAN_OR_OTHER_PACIFIC_ISLANDER
WHITE
NA
G1
G2
G3
GX
FEMALE
MALE
C
Strata
MUC16_mutant
MUC16_WT
p = 0.051
D
CSMD1_mutant
CSMD1_WT
p = 0.054
E
FAT4_mutant
FAT4_WT
p = 0.033
F
TP53_mutant
TP53_WT
p = 0.79
G
TTN_mutant
TTN_WT
p = 0.35

Figure S18

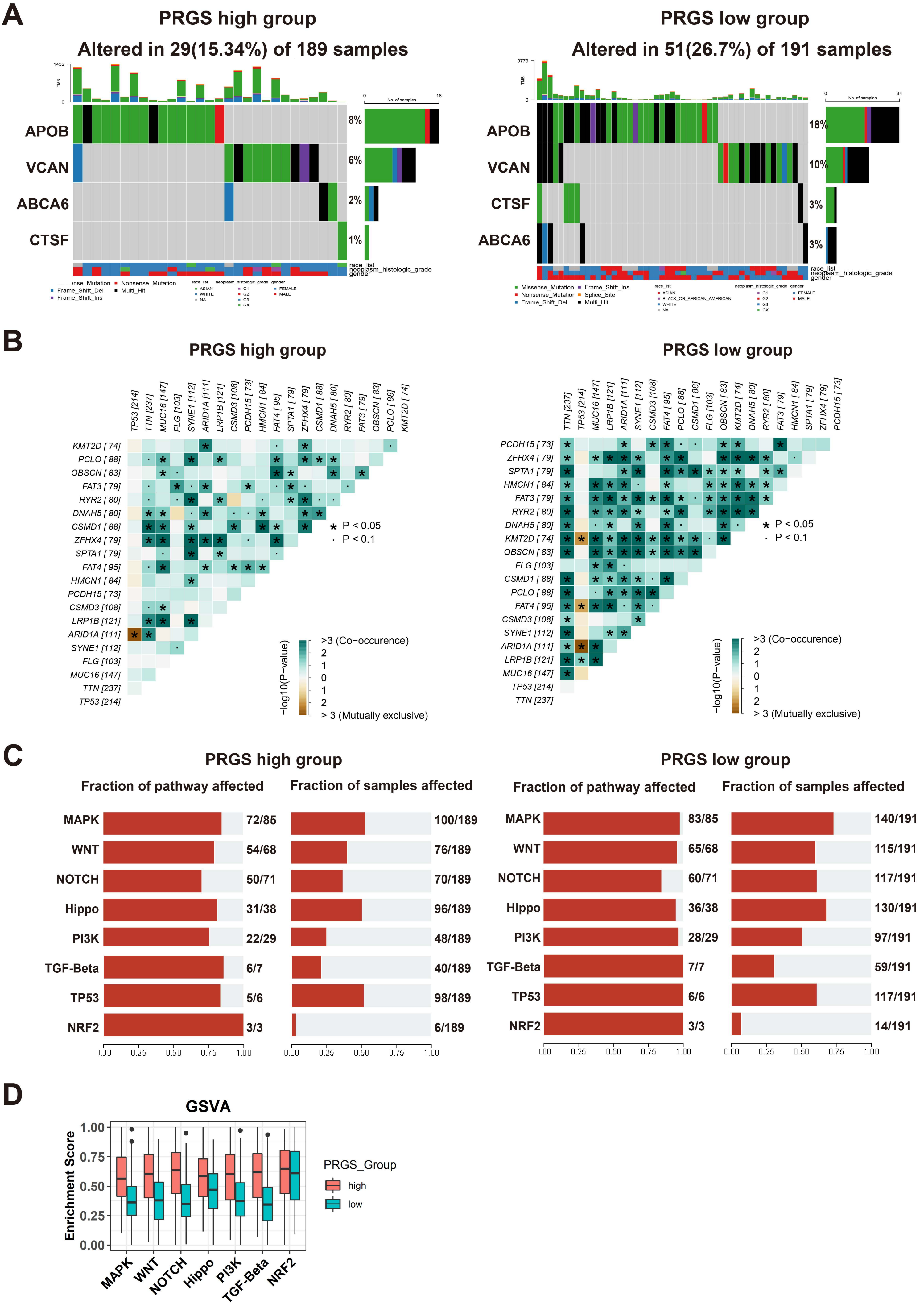
A
PRGS high group
Altered in 29(15.34%) of 189 samples
APOB
VCAN
ABCA6
CTSF
8%
6%
2%
1%
PRGS low group
Altered in 51(26.7%) of 191 samples
APOB
VCAN
CTSF
ABCA6
18%
10%
3%
3%
Missense_Mutation
Nonsense_Mutation
Frame_Shift_Del
Frame_Shift_Ins
Splice_Site
Multi_Hit
B
PRGS high group
PRGS low group
P < 0.05
P < 0.1
>3 (Co−occurence)
> 3 (Mutually exclusive)
−log10(P−value)
C
PRGS high group
Fraction of pathway affected
Fraction of samples affected
MAPK 72/85 100/189
WNT 54/68 76/189
NOTCH 50/71 70/189
Hippo 31/38 96/189
PI3K 22/29 48/189
TGF-Beta 6/7 40/189
TP53 5/6 98/189
NRF2 3/3 6/189
PRGS low group
Fraction of pathway affected
Fraction of samples affected
MAPK 83/85 140/191
WNT 65/68 115/191
NOTCH 60/71 117/191
Hippo 36/38 130/191
PI3K 28/29 97/191
TGF-Beta 7/7 59/191
TP53 6/6 117/191
NRF2 3/3 14/191
D
GSVA
Enrichment Score
PRGS_Group
high
low
MAPK
WNT
NOTCH
Hippo
PI3K
TGF-Beta
NRF2
